# KDM3A/Ets1/MCAM axis promotes growth and metastatic properties in Rhabdomyosarcoma

**DOI:** 10.18632/genesandcancer.200

**Published:** 2020

**Authors:** Lays Martin Sobral, Marybeth Sechler, Janet K. Parrish, Tyler S. McCann, Kenneth L. Jones, Joshua C. Black, Paul Jedlicka

**Affiliations:** ^1^ Department of Pathology, University of Colorado Denver, Anschutz Medical Campus, Aurora, CO, USA; ^2^ Cancer Biology Graduate Program, University of Colorado Denver, Anschutz Medical Campus, Aurora, CO, USA; ^3^ Department of Pediatrics, University of Colorado Denver, Anschutz Medical Campus, Aurora, CO, USA; ^4^ Department of Pharmacology, University of Colorado Denver, Anschutz Medical Campus, Aurora, CO, USA

**Keywords:** pediatric cancer, rhabdomyosarcoma, KDM3A, Ets1, metastasis

## Abstract

Rhabdomyosarcoma (RMS) is the most common soft tissue malignancy of childhood. RMS exists as two major disease subtypes, with oncofusion-positive RMS (FP-RMS) typically carrying a worse prognosis than oncofusion-negative RMS (FN-RMS), in part due to higher propensity for metastasis. Epigenetic mechanisms have recently emerged as critical players in the pathogenesis of pediatric cancers, as well as potential new therapeutic vulnerabilities. Herein, we show that the epigenetic regulator KDM3A, a member of the Jumonji-domain histone demethylase (JHDM) family, is overexpressed, potently promotes colony formation and transendothelial invasion, and activates the expression of genes involved in cell growth, migration and metastasis, in both FN-RMS and FP-RMS. In mechanistic studies, we demonstrate that both RMS subtypes utilize a KDM3A/Ets1/MCAM disease-promoting axis recently discovered in Ewing Sarcoma, another aggressive pediatric cancer of distinct cellular and molecular origin. We further show that KDM3A depletion in FP-RMS cells inhibits both tumor growth and metastasis *in vivo*, and that RMS cells are highly sensitive to colony growth inhibition by the pan-JHDM inhibitor JIB-04. Together, our studies reveal an important role for the KDM3A/Ets1/MCAM axis in pediatric sarcomas of distinct cellular and molecular ontogeny, and identify new targetable vulnerabilities in RMS.

## INTRODUCTION

Rhabdomyosarcoma (RMS), a malignant neoplasm of mesenchymal origin with skeletal muscle differentiation, is the most common soft tissue malignancy of childhood and young adults [1]. Biologically, RMS consists of two predominant and distinct disease subtypes [2-4]. Fusion-negative RMS (FN-RMS; typically of “embryonal” histology or “ERMS”) usually affects younger children, and occurs in more axial locations (most commonly the head/neck and genitourinary tract). Molecularly, FN-RMS is a heterogeneous disease, with frequent mutations in receptor tyrosine kinase (RTK)/Ras/PI3K signaling axes, and less common mutations in other known cancer-associated pathways. Fusion-positive RMS (FP-RMS; typically of “alveolar” histology or “ARMS”), on the other hand, usually affects older children, and occurs in more peripheral locations (most commonly arms and legs). FP-RMS, as its name implies, is driven by fusion oncogenes. These consist of in-frame fusions of the amino terminus of PAX3 or PAX7, and the carboxy terminal portion of FOXO1. PAX3 and PAX7 are transcription factors involved in normal myogenesis, and supply intact, functional DNA-binding domains to the respective oncofusions, while FOXO1 (formerly FKHR), a multifunctional transcription factor, provides a potent transcriptional activation domain.

Clinically, RMS substratifies into low, intermediate and high-risk disease, largely based on the extent (localized versus disseminated) and type (FN-RMS versus FP-RMS) of disease [1, 3, 4]. Localized FN-RMS constitutes low-risk disease, and is associated with ~80% 5-year survival. Disease spread to lymph nodes or presence of a PAX/FOXO1 oncofusion increases risk of poor outcome (intermediate-risk disease). Metastatic RMS constitutes high-risk disease, associated with <30% 5-year survival. Current treatment protocols consist of multimodal conventional chemotherapy, surgery, and, as appropriate, radiation for additional local disease control. These have not changed substantively since the 1970s, and are poorly effective in patients with high-risk disease. Identification and development of new and better treatment options for high-risk RMS is thus a pressing unmet need. Toward this goal, better understanding of molecular pathways contributing to RMS progression, and of the pathogenesis of the more aggressive FP-RMS in general, is needed.

There is growing evidence that epigenetic mechanisms play critical roles in the pathogenesis of pediatric cancers, which tend to be less genetically complex than their adult disease counterparts. Our own recent studies identified the epigenetic regulator KDM3A (JMJD1A/JHDM2A), a member of the Jumonji C domain-containing histone demethylase (JHDM) family [5, 6], as a disease-promoting factor in a pediatric sarcoma. Specifically, we showed that, in Ewing Sarcoma, an aggressive pediatric cancer of bone and soft tissue, KDM3A acts as both a tumor promoter [7], and, through its downstream-induced gene MCAM, a promoter of metastasis [8]. KDM3A has also been shown to be upregulated in expression, and to be involved in disease-promoting properties, in numerous other cancers (as recently reviewed in [9]). This, along with its restricted expression and relative paucity of essential functions, has pointed to KDM3A as a potentially attractive therapeutic target [10]. Our previous studies noted robust expression of KDM3A in RMS cell lines [7], prompting further investigation into its potential role(s), as a disease-promoting factor and candidate therapeutic target, in RMS.

## RESULTS

### KDM3A is overexpressed, and promotes colony growth and transendothelial invasion, in FN-RMS and FP-RMS

**Figure 1:**
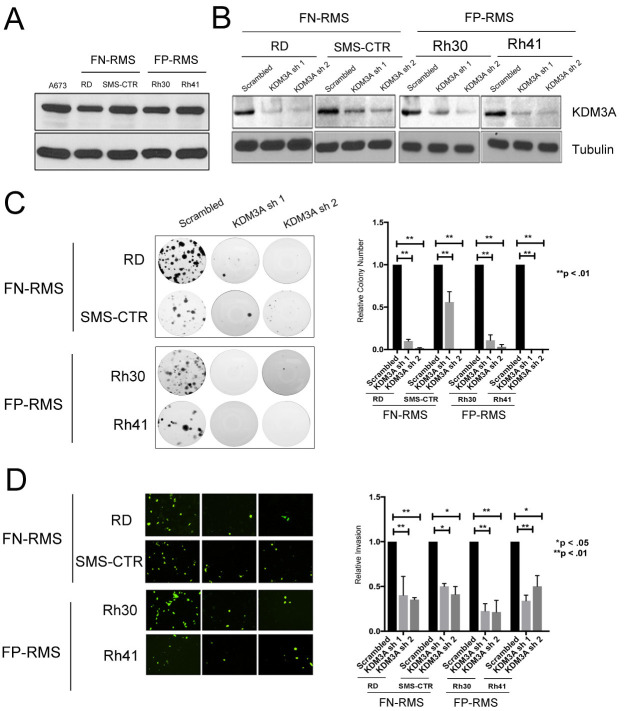
KDM3A is expressed in FN-RMS and FP-RMS patient-derived cell lines, and promotes colony formation and transendothelial invasion **A**. KDM3A protein expression in FN-RMS (RD and SMS-CTR) and FP-RMS (Rh30 and Rh41) cell lines relative to the Ewing Sarcoma A673 cells, as determined by immunoblotting. **B**. Stable, shRNA-mediated, depletion of KDM3A protein expression in RMS cells as determined via protein immunoblotting. **C**. Effects of KDM3A stable depletion on colony formation. Shown are representative images of colonies formed, and quantifications of colony count data. The latter are plotted as mean and standard error of the mean of 3 independent experiments, each performed in triplicate, with the control set to 1; p-values were determined using one-way analysis of variance (1-way ANOVA) with multiple comparisons. **D**. Effects of KDM3A stable depletion on transendothelial invasion. Shown are representative images, and quantifications, of invaded cells. Data show mean and standard error of the mean of 3 independent experiments, each performed in duplicate, with the control set to 1; p-values were determined using 1-way ANOVA with multiple comparisons.

As mentioned above, our own prior studies noted high KDM3A expression in RMS cell lines [7]. Notably, KDM3A can also be seen to be overexpressed in both FN-RMS and FP-RMS tumors relative to non-neoplastic skeletal muscle tissue in previously published gene expression profiling studies [11], and in the St Jude Children’s Research Hospital Pediatric Cancer (PeCan) database (https://pecan.stjude.cloud) (Supplementary Figure S1). To further investigate the potential role of KDM3A in RMS, we first confirmed and expanded our previous observations of KDM3A expression in multiple validated RMS patient-derived cells lines. As shown in Figure 1A, KDM3A is highly expressed in the FN-RMS lines RD and SMS-CTR, and the FP-RMS lines Rh30 and Rh41, at levels similar Ewing Sarcoma (A673 cells shown as a reference; [8]). Having confirmed high-level expression in RMS, we next evaluated KDM3A function using stable shRNA-mediated KDM3A depletion, confirmed by immunoblotting, in each of the above FN-RMS and FP-RMS cell lines (Figure 1B).

To probe potential roles of KDM3A in RMS progression, we turned to assays evaluating properties related to sarcoma growth and dissemination. For evaluation of growth properties, we used a clonogenic assay, which assesses the ability of cancer cells to initiate and expand colonies, similar to our previous studies in Ewing Sarcoma [7]. This analysis demonstrated dramatic inhibition of clonogenic growth upon KDM3A knockdown, relative to the shRNA Scramble control, in both FN-RMS RD and SMS-CTR cells, and FP-RMS Rh30 and Rh41 cells (Figure 1C). To evaluate a potential role of KDM3A in sarcoma dissemination, we used a transendothelial invasion assay, which assesses the ability of cells to traverse endothelium during the metastatic steps of vascular intravasation and extravasation. In all four cell lines, KDM3A knockdown resulted in a dramatic reduction of the ability of RMS cells to traverse an endothelial monolayer, relative to shRNA Scramble control (Figure 1D). Thus, KDM3A promotes colony growth and transendothelial invasion, important properties of disease progression, in both FN-RMS and FP-RMS.

### KDM3A positively controls pro-growth and pro-metastatic gene expression programs in FN-RMS and FP-RMS

**Figure 2:**
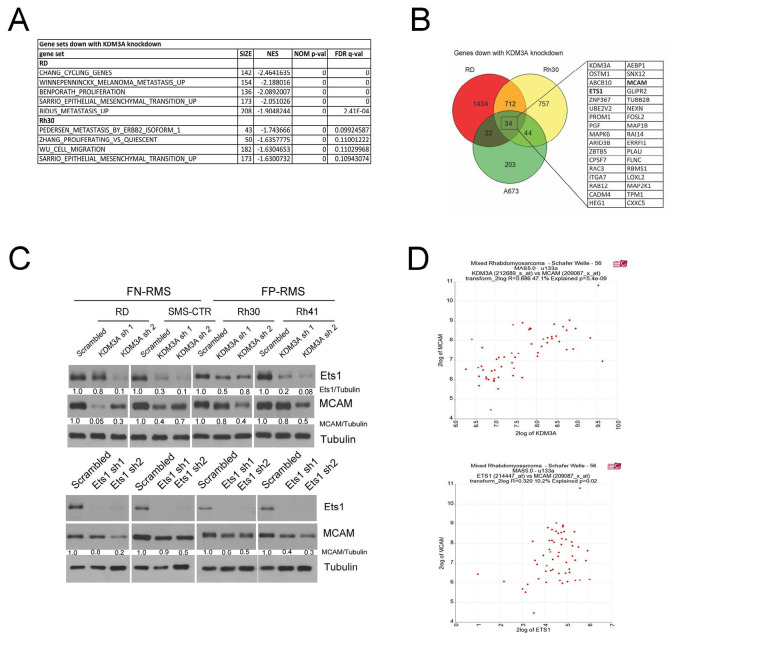
KDM3A positively controls pro-growth and pro-metastasis gene expression programs in FN-RMS and FP-RMS. **A**. Gene Set Enrichment Analysis of KDM3A transcriptomes identifies positive regulation of pro-growth and pro-metastatic programs in RD and Rh30 cells. **B**. Overlap analysis for genes subject to positive regulatory control by KDM3A in RD and Rh30 RMS cells, and Ewing Sarcoma A673 cells [8]. Genes in table on right correspond to genes shared by all three groups. **C**. Ets1 and MCAM protein expression in control and KDM3A knockdown FN-RMS and FP-RMS cells (top panels), and MCAM expression in FN-RMS and FP-RMS cells following Ets1 knockdown (bottom panels). Western blot data from representative experiments; quantifications represent mean expression levels from 3 independent experiments, as determined by densitometry, normalized to tubulin. **D**. Correlation of KDM3A and MCAM (top) and Ets1 and MCAM (bottom) expression levels in RMS patient tumors (data from R2 OncoGenomics database; http://hgserver1.amc.nl/cgi-bin/r2/main.cgi).

To further investigate the molecular roles and mechanisms of KDM3A in RMS, we used RNA-seq analysis to define the KDM3A-controlled transcriptomes in FN-RMS RD cells and FP-RMS Rh30 cells, each stably transduced with Scrambled shRNA or KDM3A-targeting shRNA. RNA-seq revealed 2296 upregulated and 2202 downregulated genes in RD cells (Q < 0.05), and 1462 upregulated and 1547 downregulated genes in Rh30 cells (Q < 0.05), upon stable KDM3A knockdown (Supplementary Table 1). In order to identify gene groups and processes of biological interest, the transcriptome data were subjected to Gene Set Enrichment Analysis (GSEA), which showed, among genes down with KDM3A knockdown in both RD and Rh30 cells, enrichment of genes sets involved in processes related to growth (proliferation, cell cycle) and dissemination (migration, epithelial-mesenchymal transition, metastasis) (Figure 2A). Thus, consistent with the phenotypes observed in our functional studies, KDM3A positively controls expression of pro-growth and pro-metastatic genes in both FN-RMS and FP-RMS cells. Given the similarity of the RMS phenotypic and transcriptomic findings to our previous studies in Ewing Sarcoma [8], we next examined the KDM3A-controlled transcriptomes in RD and Rh30 cells for specific gene overlaps with our previously defined KDM3A-controlled transcriptome in Ewing Sarcoma A673 cells [8]. This revealed 34 genes commonly upregulated by KDM3A (down with KDM3A knockdown) in FN-RMS RD cells, FP-RMS Rh30 cells, and Ewing Sarcoma A673 cells (Figure 2B). This shared group included the transcription factor Ets1 and the cell surface protein MCAM (Figure 2B), which our previous studies defined as part of a novel, disease-promotional axis in Ewing Sarcoma [8].

### Ets1 contributes to positive regulatory control of MCAM by KDM3A, in FN-RMS and FP-RMS

Our previous studies in Ewing Sarcoma showed that KDM3A positively controls the expression of Ets1, and that KDM3A and Ets1 both control expression of MCAM [8]. To determine whether a similar regulatory relationship holds true in RMS, we examined the effects of KDM3A and Ets1 depletion on Ets1 and MCAM expression in our RMS cell lines. Similar to our prior Ewing Sarcoma studies, this revealed that KDM3A controls both Ets1 and MCAM expression, and Ets1 also controls MCAM expression, in both FN-RMS and FP-RMS (Figure 2C). RD and SMS-CTR cells each have an activating Ras mutation [12], and Ets1 is a known nuclear effector of Ras/MAPK signaling [13]. To determine whether regulation of MCAM by Ets1 in FN-RMS is dependent on constitutively active Ras signaling, we examined the effects of Ets1 on MCAM expression in Ras^WT^ Rh18 FN-RMS cells. Ets1 depletion decreased MCAM levels in Rh18 cells (Supplementary Figure S3), indicating that Ets1 regulation of MCAM in FN-RMS extends to RasWT disease. Moreover, and in further support of the broad relevance of these regulatory relationships in RMS, examination of public gene expression profiling data showed that KDM3A and Ets1 each significantly correlate with MCAM expression in RMS patient tumors (R2 OncoGenomics database; Figure 2D).

**Figure 3:**
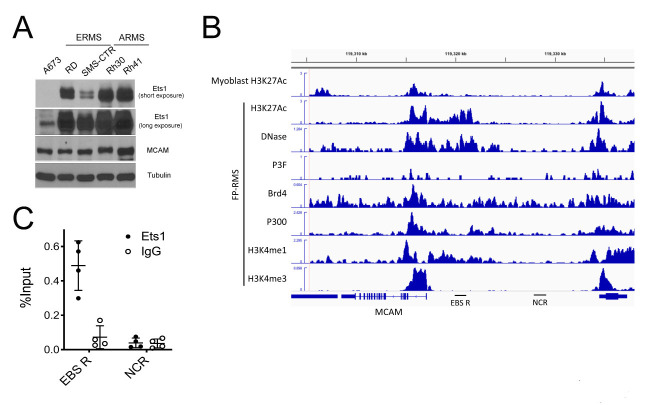
**A**. Relative Ets1 protein expression in FN-RMS and FP-RMS cells, and Ewing Sarcoma A673 cells as determined by immunoblotting. **B**. Cistrome data at the MCAM genomic locus (from CistromeDB; http://cistrome.org/db), visualized in the Integrated Genomics Viewer (IGV). Myoblast H3K27Ac data from [32]; FP-RMS cistrome data from [14] (H3K27Ac data from Rh5 cells; all other data from Rh4 cells); Ets1 binding site region (EBS R) denotes genomic locus containing 4 candidate Ets1 binding sites (Supplemental Figure S4), interrogated in ChIP analyses in “C”; NCR: negative control region used for ChIP analyses; P3F: PAX3/FOXO1. C. ChIP-qPCR data from 4 independent experiments interrogating EBS R and NCR with Ets1 and negative control (IgG) antibody (mean and SD of % input).

We noted Ets1 expression to be dramatically higher in RMS cell lines relative to Ewing Sarcoma A673 cells, in which we had originally identified Ets1 as an important component of the KDM3A/Ets1/MCAM disease-promoting axis [8] (Figure 3A). Moreover, we found Ets1 protein levels to be overall higher in FP-RMS relative to FN-RMS (Figure 3A). Our previous studies showed that Ets1 directly controls MCAM expression in Ewing Sarcoma. Recent studies performed detailed characterization of the regulatory cistrome in FP-RMS [14]. Examination of these data showed an active regulatory region at the MCAM genomic locus in FP-RMS, including: increased DNase hypersensitivity; elevated levels of H3K27Ac, H3K4me3, and presence of the H3K4me1 enhancer mark; and possible association of the P300 and Brd4 coactivators (Figure 3B, boxed region; no significant enrichment of PAX3/FOXO1 (P3F) binding is observed at the MCAM genomic locus, consistent with MCAM not being identified as a direct P3F target [14]). Interrogation of this region by Ets1 chromatin immunoprecipitation (ChIP) in FP-RMS Rh41 cells revealed robust and reproducible enrichment over a locus also containing multiple Ets1 DNA response elements (“EBS R”; Figure 3C and Supplementary Figure S4), thus supporting a direct mechanism of regulation of MCAM expression by Ets1. Interrogation of this same region by KDM3A ChIP did not reveal evidence of direct association, nor were we able to demonstrate evidence of KDM3A association with the Ets1 promoter (data not shown). Thus, in contrast to our previous findings in Ewing Sarcoma, KDM3A does not appear to control Ets1 and MCAM expression via direct association with proximal regulatory elements in FP-RMS.

### MCAM strongly phenocopies effects of KDM3A on colony growth and transendothelial invasion, in FN-RMS and FP-RMS

**Figure 4:**
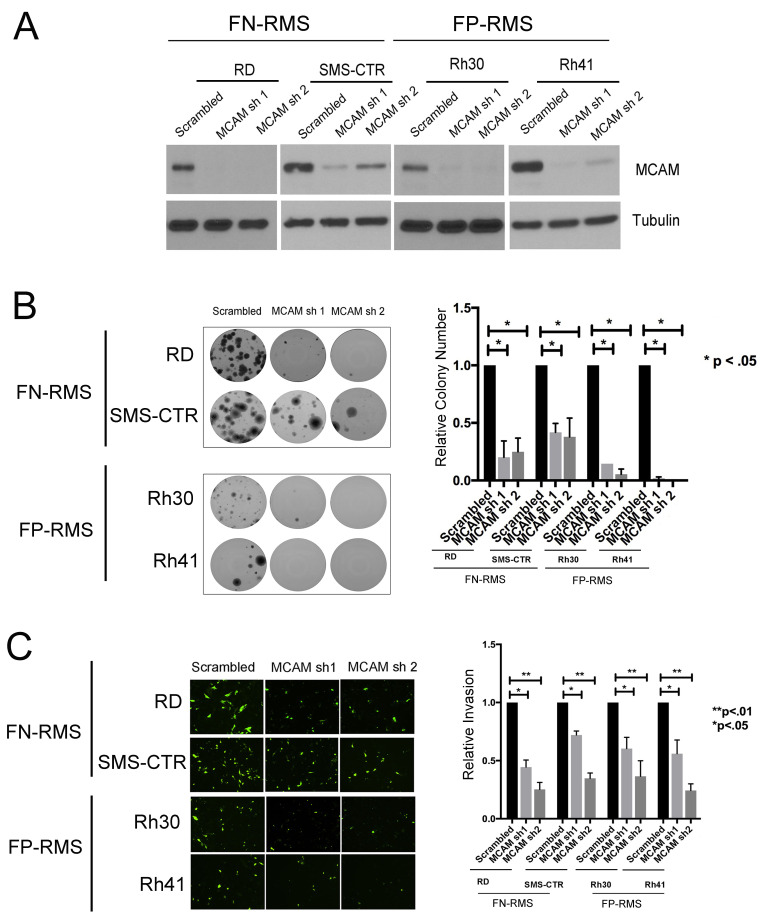
MCAM promotes colony growth and transendothelial invasion in FN-RMS and FP-RMS cells. **A**. MCAM knockdown as determined by immunoblotting. **B**. Clonogenic and **C**. transendothelial invasion assays in control and MCAM knockdown cells. Representative images and quantifications are shown. Quantifications represent mean and standard error of the mean of 3 independent experiments, each performed in triplicate, with control set to 1; p-values were determined using 1-way ANOVA with multiple comparisons.

MCAM has previously been identified as a prevalently overexpressed cell surface protein in pediatric cancers [15], and our own prior studies demonstrated it to be an important mediator of KDM3A effects in Ewing Sarcoma [8]. To determine whether the role of MCAM as a mediator of KDM3A effects is conserved in FN-RMS or/and FP-RMS, we examined the phenotypic effects of shRNA-mediated MCAM depletion in our RMS cell line panel (Figure 4A). MCAM depletion in both FN-RMS RD and SMS-CTR and FP-RMS Rh30 and Rh41 cell lines resulted in potent inhibition of colony formation in the clonogenic assay (Figure 4B), as well as transendothelial invasion (Figure 4C). Thus, MCAM potently promotes both growth and invasive properties of FN-RMS and FP-RMS cells, and strongly phenocopies the effects of KDM3A in both RMS subtypes. These findings support a role for MCAM as an important mediator of KDM3A action in both subtypes of RMS, similar to our previous findings in Ewing Sarcoma [8].

### KDM3A promotes tumor growth and metastasis in FP-RMS xenograft models *in vivo*

**Figure 5:**
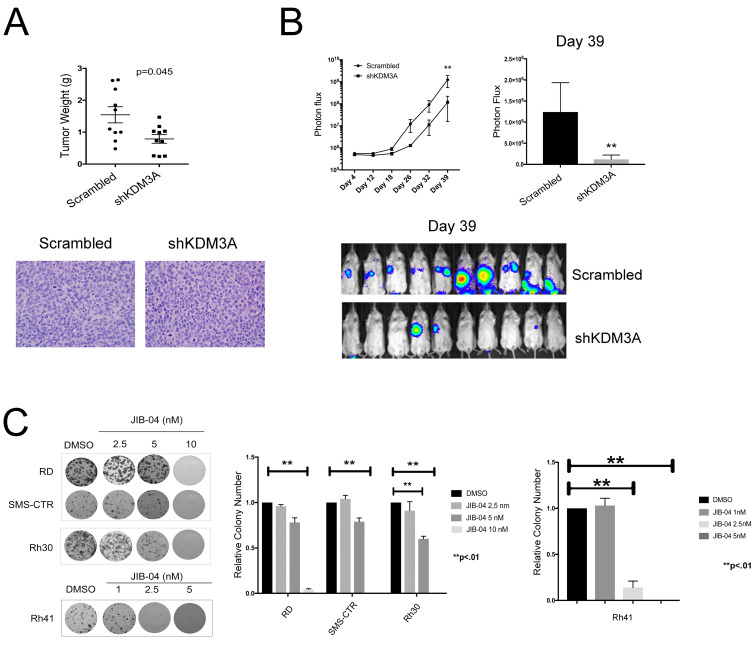
*In vivo* xenograft and pharmacologic inhibitor studies. **A**. KDM3A depletion inhibits tumor growth in an orthotopic gastrocnemius injection xenograft model. 2 x 10^6^ Scramble control or shKDM3A (sh2) FP-RMS Rh30 cells were injected into the gastrocnemius muscle of immunocompromised (NOD-SCID/Gamma) mice (10 animals/group). Tumor weights (individual values, mean and standard error) at necropsy (day 25) are shown; p-value was determined using a two-tailed Mann-Whitney test. Tumors from both groups were characterized by malignant round and spindle cells with variable amounts of eosinophilic cytoplasm, characteristic of RMS (images below, H+E histology, 40x magnification). **B**. KDM3A depletion decreases metastasis in a tail vein injection model. 1 x 10^6^ Scramble control or shKDM3A (sh2) Rh30 cells, each additionally expressing a luciferase reporter, were injected into the tail vein of NOD-SCID/Gamma mice (10 animals/group). Metastasis development was monitored weekly using IVIS imaging following administration of luciferin. Left panel shows data from full experimental time course (mean and standard error of photon flux), plotted on a log scale (**: p = 0.001, using 2-way ANOVA with repeated measures); right panel shows the same data for the last time point (day 39), plotted on a linear scale, along with corresponding IVIS images below. **C**. JIB-04 treatment potently inhibits colony growth of FN-RMS and FP-RMS cells. Beginning one day after plating, JIB-04 or vehicle control (DMSO) was added at the indicated concentration, and replaced every 3 days for 15 total days, at which point colonies were stained and quantified as in Figure 1. Representative images from one experiment, and colony quantifications from 2 independent experiments, each performed in duplicate, are shown; data are plotted as mean and standard error, with control set to 1; p-values were determined using 1-way ANOVA with multiple comparisons (no colonies were observed in SMS-CTR and Rh30 cells treated with 10 nM JIB-04, and in Rh41 cells treated with 5 nM JIB-04).

KDM3A transcriptome analysis revealed, in addition to Ets1 and MCAM, other genes previously implicated in the promotion of aggressive cancer properties, including the genes FYN, AXL, LOXL2 and PLAU [16-18]. We confirmed the regulation of FYN by KDM3A in both FN-RMS and FP-RMS cell lines and of AXL, LOXL2 and PLAU in both FP-RMS cell lines (Supplementary Figure S5). Together, the above findings suggest an important role for KDM3A in RMS disease progression. To further evaluate the role of KDM3A in RMS, we examined the effects of its depletion in animal xenograft models of the disease, focusing on the more aggressive FP-RMS disease subtype. To evaluate the role of KDM3A in tumor growth, we employed an orthotopic gastrocnemius injection model in NOD-SCID/Gamma mice. In this model, stable depletion of KDM3A in the FP-RMS Rh30 cell line resulted in significantly smaller tumors compared to the Scramble shRNA control (Figure 5A), thus confirming, *in vivo*, the findings of our *in vitro* colony formation studies. To evaluate the role of KDM3A in metastasis, we employed a tail vein injection experimental metastasis model, also in NOD-SCID/Gamma mice. In this model, stable depletion of KDM3A in the FP-RMS Rh30 cell line resulted in a significantly smaller metastatic disease burden (Figure 5B), thus supporting a role for KDM3A in metastasis promotion *in vivo*. Based on the phenotypic analyses *in vitro* (Figure 1), it is likely that the reduced metastatic burden upon KDM3A depletion is an aggregate effect of diminished growth and invasive properties.

### The pan-JHDM pharmacologic inhibitor JIB-04 potently inhibits colony growth in FN-RMS and FP-RMS

Specific pharmacologic inhibitors of KDM3A do not exist at this time. However, our recent studies demonstrated growth-inhibitory activity of a pan-JHDM inhibitor (JIB-04 [19]), in Ewing Sarcoma [20]. To determine whether JIB-04 also inhibits the growth of RMS cells, we examined its effects in the clonogenic assay. Treatment of FN-RMS and FP-RMS cell lines with JIB-04 resulted in potent inhibition of clonogenic growth at low nanomolar concentrations, with particularly strong effects in the FP-RMS cells, especially Rh41 cells (Figure 5C). Thus, similar to our previous findings in Ewing Sarcoma, JIB-04 inhibits RMS colony growth.

## DISCUSSION

Our previous studies identified a new regulatory axis with growth and metastasis promotional properties, involving KDM3A, Ets1 and MCAM, in Ewing Sarcoma [7, 8]. In the current studies, we show that this axis is functionally conserved in both FN-RMS, and the, typically more aggressive, FP-RMS.

Ewing Sarcoma is an aggressive, poorly differentiated pediatric neoplasm most commonly arising in bone, but also soft tissue and other sites [21]. Ewing Sarcoma pathogenesis is driven by EWS/Ets, most commonly EWS/Fli1, fusion oncoproteins [22, 23]. The definitive cell of Ewing Sarcoma origin remains undefined, but best available evidence points to mesenchymal or neural crest stem cells as the likely disease source [24, 25]. Similar to Ewing Sarcoma, the precise cellular ontogeny of RMS has been extensively investigated. In keeping with the myogenic differentiation pathognomonic of RMS, most studies point to cells along the pathway of skeletal muscle differentiation as the likely source of both FN-RMS and FP-RMS [4, 26], although, interestingly, FN-RMS can also arise in non-myogenic cells [27]. As noted above, FN-RMS is a molecularly heterogeneous disease with diverse drivers including, most commonly, mutations in RTK/Ras signaling pathways, while FP-RMS is driven by PAX3/7-FOXO1 fusion oncoproteins [2, 3]. Ewing Sarcoma, FN-RMS and FP-RMS thus represent neoplastic diseases of distinct cellular and molecular ontogeny. In this light, it is noteworthy that a similar KDM3A/Ets1/MCAM disease-promoting axis operates in all three malignancies. It will be interesting to see whether all or portions of this axis might be more generally utilized by other sarcomas.

Although broadly conserved in terms of overall regulatory relationships and function, our studies also suggest that there are differences in specific mechanisms of regulation, namely how KDM3A controls Ets1 and MCAM expression. Our studies in Ewing Sarcoma and FP-RMS identify Ets1 as a direct regulator of MCAM expression. However, our prior studies in Ewing Sarcoma also demonstrated KDM3A localization to the Ets1 and MCAM promoter regions, while our current analogous studies in FP-RMS did not. Our FP-RMS studies suggest that KDM3A either controls Ets1 and MCAM expression indirectly, or, alternatively, through more remote regulatory elements (ie: distal enhancers). An important role for the latter in FP-RMS has recently been demonstrated [14]. Further definition of the KDM3A RMS cistrome will assist in answering these questions.

Ewing Sarcoma and FP-RMS are emerging as particularly epigenetically driven diseases, likely exemplifying a more general molecular pathogenic paradigm in transcription factor oncofusion-driven sarcomagenesis. Epigenetic mechanisms characterized in FP-RMS at this point include those involving the chromatin factors BRD4, CHD4, EZH2 and JARID2 [11, 14, 28, 29], as well as utilization of myogenic transcription factor networks [14]. Our studies add the chromatin factor KDM3A and the Ets1 transcription factor to this list of functionally important molecular components of FP-RMS pathogenesis/progression. Further understanding of how these and other epigenetic mechanisms interface with PAX/FOXO1 oncofusions, and with one another, can be expected to illuminate key aspects of the molecular basis of FP-RMS pathogenesis, as well as unlock novel approaches to inhibition of PAX/FOXO1 disease-driving action, as recently demonstrated for BRD4 [14].

KDM3A and MCAM each present potential new therapeutic targets in both subtypes of RMS, similar to our previous findings in Ewing Sarcoma [7, 8]. Strategies to inhibit KDM3A may be particularly attractive, as we show that it controls the expression of not just the Ets1/MCAM axis, but other genes expected to contribute to aggressive disease biology, such as FYN, AXL, LOXL2 and PLAU, especially in FP-RMS. KDM3A-specific inhibitors do not currently exist, but our studies present evidence of efficacy of the pan-JHDM inhibitor JIB-04 in RMS. The mechanisms of action of JIB-04 in RMS remain to be clarified, and, given its broad spectrum of anti-JHDM activity, are likely to be complex. Interestingly, this inhibitor shows greater activity in FP-RMS cells, consistent with greater dependence on epigenetic mechanisms in this RMS subtype [14]. Our findings support further evaluation of JIB-04 action in FP-RMS, and potentially other fusion-driven sarcomas. Notably, since its identification only a few years ago, JIB-04 has at this point been shown to have activity against a number of different cancers, including chemoresistant disease (as recently reviewed [9]).

In summary, we show that the KDM3A/Ets1/MCAM molecular axis, which we have previously demonstrated to manifest tumor and metastasis promotional properties in Ewing Sarcoma, also plays potent disease-promoting roles in both FN-RMS and FP-RMS. Our findings further suggest that KDM3A and MCAM, the pharmacologically targetable components of this axis, merit further attention as potential new therapeutic targets in all three diseases.

## MATERIALS AND METHODS

### Cell lines

A673 cells and culture conditions have been previously described [30]. RD and Rh30 cells were obtained from the American Tissue Culture Collection (ATCC). SMS-CTR, Rh41 and Rh18 cells were kindly provided by Dr. Mark Hatley of St. Jude Children’s Research Hospital. All cell lines were authenticated at our institution by STR profiling, and repeatedly verified to be Mycoplasma-free. Cells were grown in either DMEM (RD and SMS-CTR) or RPMI (Rh30, Rh41 and Rh18), with 10% fetal bovine serum, 1% Penicillin/Streptomycin (all cell lines except Rh18), 10 mM Hepes, 1x MEM non-essential amino acids and 1 mM Sodium Pyruvate, in a humidified atmosphere of 5% CO_2_ at 37oC.

### Stable depletion of gene expression

Stable, shRNA-mediated, depletion of KDM3A, Ets1 and MCAM expression in RMS cells was performed as previously described [30] using control, non-targeting, Scrambled shRNA (Addgene plasmid 1864), and shRNAs targeting KDM3A, Ets1 and MCAM described previously [8]. Cells were selected with 1 µg/ml of Puromycin for 3-4 days, and depletion of gene expression was verified using protein immunoblotting.

### Protein immunoblotting

Protein immunoblotting was performed as previously described [7, 8, 30]. Primary antibodies used were: KDM3A (ProMab Biotechnologies; #30134; 1:1,000); Ets1 (Cell Signaling; #14069; 1:1,000); MCAM (Proteintech; #17564-1-AP; 1:1,000); FYN (Cell Signaling, #4023; 1:1,000); tubulin (Sigma; T5168; 1:20,000).

### Quantification of RNA expression

Cells were harvested at 70-80% confluence in TRIzol (Invitrogen), and RNA was extracted per manufacturer’s instructions. RNA levels of specific transcripts were assessed by qRT-PCR (using qScript Super Mix and Perfecta SYBR Green Fast Mix; Quantabio) with RPL19 RNA as the internal control (primers are listed in Supplementary Table 2).

### Growth assays

For clonogenic growth assays, following shRNA transduction and selection, cells were plated at 700 cells (RD, SMS-CTR and Rh30) or 1,000 cells (Rh41) per well in 6-well plates. Media were changed once a week. Colonies were stained 15 days later with 0.1% crystal violet in 25% MeOH and quantified using the Image J software, as previously described [8]. MTT assays were performed as previously described [31], plating 5,000 cells per well in 96-well plates.

### Transendothelial invasion assay

Transendothelial invasion assays were performed using a Boyden chamber and a monolayer of human umbilical vein endothelial cells (HUVECs, obtained from Lonza), maintained in EBM-Plus Endothelial Cell Growth Basal Medium Plus (CC-5036, Lonza). To prepare the assay, 2 x 10^5^ HUVECs were plated in the top of the well insert (8µm pore, BD Biosciences, #353097), and inserts were placed in a 24-well companion plate with 500 µl of EBM media. Following HUVEC attachment and formation of a confluent monolayer (~6 hours), EBM media was removed and the HUVEC monolayer was washed 2 times with PBS, to remove unattached cells. The indicated RMS cells, transduced with Scrambled control or KDM3A-targeting shRNA, were harvested, counted and stained with Calcein AM vital dye (C1430, ThermoFisher Scientific). 5 x 10^4^ cells were then plated in 200 µl of serum-free media on top of the HUVEC cell monolayer, and the inserts were placed in a 24-well plate with 500 µl of media containing 5% fetal bovine serum (FBS) as a chemoattractant. After 16 hours (chosen as a time interval during which significant effects of knockdown on cell growth/survival were not observed; Supplementary Figure S2), uninvaded cells and HUVECs were removed by cleaning the top of the membrane with a cotton swab. Invaded cells on the bottom of the membrane were visualized using a fluorescence microscope, and five random fields at 10x power were imaged, and quantified using Image J software.

### JHDM inhibitor studies

Cells (700 per well for RD, SMS-CTR and Rh30 experiments; 1000 per well for Rh41 experiments) were plated in 6-well plates. Beginning one day after plating, JIB-04 (ApexBio, dissolved in DMSO) was added and replaced every 3 days for 15 total days, at which point colonies were stained and quantified.

### *in vivo* xenograft studies

For the orthotopic gastrocnemius injection xenograft model studies, 2 x 10^6^ Scramble control or shKDM3A (sh2) FP-RMS Rh30 cells were injected as a 1:1 mixture with Matrigel into the gastrocnemius muscle of immunocompromised (NOD-SCID/Gamma) mice (10 animals/group). Tumor weights were determined at necropsy (day 25). For the tail vein injection xenograft model studies, 1 x 10^6^ Scramble control or shKDM3A (sh2) Rh30 cells, each additionally expressing a luciferase reporter (described in [8]), were injected into the tail vein of NOD-SCID/Gamma mice (10 animals/group). Metastasis development was monitored weekly using IVIS imaging following administration of luciferin.

### Transcriptome analysis

Transcriptome profiling was performed on triplicate samples of FN-RMS RD/ Scramble and KDM3A-sh2 cells, and FP-RMS Rh30/ Scramble and KDM3A-sh2 cells. RNA was isolated using TRIzol (Invitrogen), and further purified using the Qiagen MinElute column kit. Samples were submitted to University of Colorado Cancer Center Microarray and Genomics shared resource for analysis of RNA quality, library preparation, and directional mRNA next-generation sequencing at 50 cycles of single-end reads on an Illumina Hi-Seq 4000 instrument. Sequencing data were processed through a custom computational pipeline consisting of the open-source gSNAP, Cufflinks and R for alignment and discovery of differential gene expression. Fragments per kilobase of exon per million mapped reads (FPKM) were used for comparison of transcript levels, and significant differences in gene expression were calculated using ANOVA in R. Deposition of the expression profiling data into the NCBI Gene Expression Omnibus database has been initiated (accession number pending). Gene Set Enrichment Analysis was performed using GSEA software (PMID: 16199517), with the KDM3A transcriptomes as the rank-ordered datasets. Gene sets with p < 0.05 (after 1000 gene set permutations) were deemed to be enriched in each group (NES: Normalized Enrichment Score; FDR: False Discovery Rate). Venn diagram analysis was performed using the on-line tool http://genevenn.sourceforge.net.

### Chromatin immunoprecipitation

Rh41 cells were cross-linked with 1% formaldehyde, followed by quenching with 0.125 M glycine, both at room temperature. Cells were washed 2x with ice-cold PBS, collected in ice-cold PBS by scraping, counted, pelleted, and resuspended in Cell Lysis Buffer (5 mM PIPES, pH 8.0; 85 mM KCl; 0.5% NP-40). Following incubation on ice for 10 minutes, a nuclear-enriched fraction was collected by centrifugation for 5 minutes at 5000 rpm at 4oC. The pellet was resupended in ChIP Lysis Buffer (50 mM Tris-HCl, pH 8.1; 10 mM EDTA; 0.2% SDS; 0.1 mM PMSF; 1µg/ml each of aprotinin and leupeptin) on ice, and subjected to sonication in the Bioruptor Plus apparatus (Diagenode) for 30 cycles (each 30 seconds on/ 30 seconds off) at high power. The resulting sonicate was centrifuged at 15,000 rpm for 10 minutes at 4°C to pellet debris. The supernatant was collected, and chromatin was quantified and stored in 10 µg aliquots at -80°C. Following verification of appropriate chromatin fragmentation, 10 µg of chromatin was diluted in 500 µl of ChIP Dilution Buffer (16.7 mM Tris-HCl, pH 8.1; 167 mM NaCl; 1.2 mM EDTA; 0.01% SDS; 1.1% Triton-X100), and pre-cleared by addition of 50 µl of protein A/G agarose beads (Thermo Scientific, #20423) and rotation for 1 hour at 4°C. Samples were spun briefly to pellet the beads. 50 µl (10%) of supernatant was set aside as Input. For ChIP, Ets1 antibody (Active Motif; #39580) or negative control IgG antibody (Cell Signaling, #2729) was added to 500 µl of the remaining pre-cleared chromatin preparation, and the samples were incubated overnight with rotation at 4°C. 20 µl of magnetic protein A/G beads (EMD Millipore, #16-663) were added, and the samples were rotated at 4°C for 4 hours. The ChIP-bead complexes were sequentially washed: 2x with low salt buffer (20 mM Tris-HCL pH 8.1, 150 mM NaCl, 2 mM EDTA, 0.1% SDS, 1% Triton-X100); 2x with high salt buffer (20 mM Tris-HCL pH 8.1, 500 mM NaCl, 2 mM EDTA, 0.1% SDS, 1% Triton-X100); 2x with LiCl buffer (10 mM Tris pH 8.1, 1 mM EDTA, 0.25 M LiCl, 1% NP-40, 1% deoxycholic acid); and 2x with TE buffer. Cross-links were reversed and ChIP DNA was recovered by: addition of 200 µl of Elution buffer (0.1 M NaHCO_3_, 1% SDS) and 0.2 M NaCl, followed by overnight incubation at 65°C; addition of 10 µg RNase A and incubation at 37°C with for 1 hour; addition of 20 µg Proteinase K and incubation at 55°C for 1 hour; and phenol/chloroform extraction and ethanol precipitation. Dry ChIP DNA was resuspended in 50 µl of H_2_0 and analyzed for enrichment of specific genomic regions, relative to Input DNA, by qPCR (primer sequences are listed in Supplementary Table 2).

## SUPPLEMENTARY MATERIAL AND TABLES





## References

[1] Ognjanovic S, Linabery AM, Charbonneau B, Ross JA (2009). Trends in childhood rhabdomyosarcoma incidence and survival in the United States, 1975-2005.. Cancer.

[2] Shern JF, Chen L, Chmielecki J, Wei JS, Patidar R, Rosenberg M, Ambrogio L, Auclair D, Wang J, Song YK, Tolman C, Hurd L, Liao H (2014). Comprehensive genomic analysis of rhabdomyosarcoma reveals a landscape of alterations affecting a common genetic axis in fusion-positive and fusion-negative tumors.. Cancer Discov.

[3] Shern JF, Yohe ME, Khan J (2015). Pediatric Rhabdomyosarcoma.. Crit Rev Oncog.

[4] Sun X, Guo W, Shen JK, Mankin HJ, Hornicek FJ, Duan Z (2015). Rhabdomyosarcoma: Advances in Molecular and Cellular Biology.. Sarcoma.

[5] Yamane K, Toumazou C, Tsukada Y, Erdjument-Bromage H, Tempst P, Wong J, Zhang Y (2006). JHDM2A, a JmjC-containing H3K9 demethylase, facilitates transcription activation by androgen receptor.. Cell.

[6] Cloos PA, Christensen J, Agger K, Helin K (2008). Erasing the methyl mark: histone demethylases at the center of cellular differentiation and disease.. Genes Dev.

[7] Parrish JK, Sechler M, Winn RA, Jedlicka P (2015). The histone demethylase KDM3A is a microRNA-22-regulated tumor promoter in Ewing Sarcoma.. Oncogene.

[8] Sechler M, Parrish JK, Birks DK, Jedlicka P (2017). The histone demethylase KDM3A, and its downstream target MCAM, promote Ewing Sarcoma cell migration and metastasis.. Oncogene.

[9] McCann TS, Sobral LM, Self C, Hsieh J, Sechler M, Jedlicka P (2019). Biology and targeting of the Jumonji-domain histone demethylase family in childhood neoplasia: a preclinical overview.. Expert Opin Ther Targets.

[10] Jedlicka P (2017). The potential of KDM3A as a therapeutic target in Ewing Sarcoma and other cancers.. Expert Opin Ther Targets.

[11] Walters ZS, Villarejo-Balcells B, Olmos D, Buist TW, Missiaglia E, Allen R, Al-Lazikani B, Garrett MD, Blagg J, Shipley J (2014). JARID2 is a direct target of the PAX3-FOXO1 fusion protein and inhibits myogenic differentiation of rhabdomyosarcoma cells.. Oncogene.

[12] Yohe ME, Gryder BE, Shern JF, Song YK, Chou HC, Sindiri S, Mendoza A, Patidar R, Zhang X, Guha R, Butcher D, Isanogle KA, Robinson CM (2018). MEK inhibition induces MYOG and remodels super-enhancers in RAS-driven rhabdomyosarcoma.. Sci Transl Med.

[13] Wasylyk B, Hagman J, Gutierrez-Hartmann A (1998). Ets transcription factors: nuclear effectors of the Ras-MAP-kinase signaling pathway.. Trends Biochem Sci.

[14] Gryder BE, Yohe ME, Chou HC, Zhang X, Marques J, Wachtel M, Schaefer B, Sen N, Song Y, Gualtieri A, Pomella S, Rota R, Cleveland A (2017). PAX3-FOXO1 Establishes Myogenic Super Enhancers and Confers BET Bromodomain Vulnerability.. Cancer Discov.

[15] Orentas RJ, Yang JJ, Wen X, Wei JS, Mackall CL, Khan J (2012). Identification of cell surface proteins as potential immunotherapy targets in 12 pediatric cancers.. Front Oncol.

[16] Rankin EB, Giaccia AJ (2016). The Receptor Tyrosine Kinase AXL in Cancer Progression.. Cancers (Basel).

[17] Wu L, Zhu Y (2015). The function and mechanisms of action of LOXL2 in cancer (Review).. Int J Mol Med.

[18] Mahmood N, Mihalcioiu C, Rabbani SA (2018). Multifaceted Role of the Urokinase-Type Plasminogen Activator (uPA) and Its Receptor (uPAR): Diagnostic, Prognostic, and Therapeutic Applications.. Front Oncol.

[19] Wang L, Chang J, Varghese D, Dellinger M, Kumar S, Best AM, Ruiz J, Bruick R, Peña-Llopis S, Xu J, Babinski DJ, Frantz DE, Brekken RA (2013). A small molecule modulates Jumonji histone demethylase activity and selectively inhibits cancer growth.. Nat Commun.

[20] Parrish JK, McCann TS, Sechler M, Sobral LM, Ren W, Jones KL, Tan AC, Jedlicka P (2018). The Jumonji-domain histone demethylase inhibitor JIB-04 deregulates oncogenic programs and increases DNA damage in Ewing Sarcoma, resulting in impaired cell proliferation and survival, and reduced tumor growth.. Oncotarget.

[21] Ludwig JA (2008). Ewing sarcoma: historical perspectives, current state-of-the-art, and opportunities for targeted therapy in the future.. Curr Opin Oncol.

[22] Sankar S, Lessnick SL (2011). Promiscuous partnerships in Ewing’s sarcoma.. Cancer Genet.

[23] Jedlicka P (2010). Ewing Sarcoma, an enigmatic malignancy of likely progenitor cell origin, driven by transcription factor oncogenic fusions.. Int J Clin Exp Pathol.

[24] Tirode F, Laud-Duval K, Prieur A, Delorme B, Charbord P, Delattre O (2007). Mesenchymal stem cell features of Ewing tumors.. Cancer Cell.

[25] von Levetzow C, Jiang X, Gwye Y, von Levetzow G, Hung L, Cooper A, Hsu JH, Lawlor ER (2011). Modeling initiation of Ewing sarcoma in human neural crest cells.. PLoS One.

[26] Kashi VP, Hatley ME, Galindo RL (2015). Probing for a deeper understanding of rhabdomyosarcoma: insights from complementary model systems.. Nat Rev Cancer.

[27] Drummond CJ, Hanna JA, Garcia MR, Devine DJ, Heyrana AJ, Finkelstein D, Rehg JE, Hatley ME (2018). Hedgehog Pathway Drives Fusion-Negative Rhabdomyosarcoma Initiated From Non-myogenic Endothelial Progenitors. Cancer Cell.

[28] Böhm M, Wachtel M, Marques JG, Streiff N, Laubscher D, Nanni P, Mamchaoui K, Santoro R, Schäfer BW (2016). Helicase CHD4 is an epigenetic coregulator of PAX3-FOXO1 in alveolar rhabdomyosarcoma.. J Clin Invest.

[29] Ciarapica R, De Salvo M, Carcarino E, Bracaglia G, Adesso L, Leoncini PP, Dall’Agnese A, Walters ZS, Verginelli F, De Sio L, Boldrini R, Inserra A, Bisogno G, The Polycomb group (2014). The Polycomb group (PcG) protein EZH2 supports the survival of PAX3-FOXO1 alveolar rhabdomyosarcoma by repressing FBXO32 (Atrogin1/MAFbx).. Oncogene.

[30] McKinsey EL, Parrish JK, Irwin AE, Niemeyer BF, Kern HB, Birks DK, Jedlicka P (2011). A novel oncogenic mechanism in Ewing sarcoma involving IGF pathway targeting by EWS/Fli1-regulated microRNAs.. Oncogene.

[31] Moore C, Parrish JK, Jedlicka P (2017). MiR-193b, downregulated in Ewing Sarcoma, targets the ErbB4 oncogene to inhibit anchorage-independent growth.. PLoS One.

[32] Consortium EP, ENCODE Project Consortium (2012). An integrated encyclopedia of DNA elements in the human genome.. Nature.

