# Supplementary material for: KDM3A/Ets1/MCAM axis promotes growth and metastatic properties in Rhabdomyosarcoma
Source: Genes Cancer. 2020;11(1-2):53–65. doi: 10.18632/genesandcancer.200 (PMC7289905; doi:10.18632/genesandcancer.200)
Supplement: Supplementary file 1 [file ganc-11-53-s001.pdf]

KDM3A/Ets1/MCAM axis promotes growth and metastatic properties in Rhabdomyosarcoma – Sobral et al

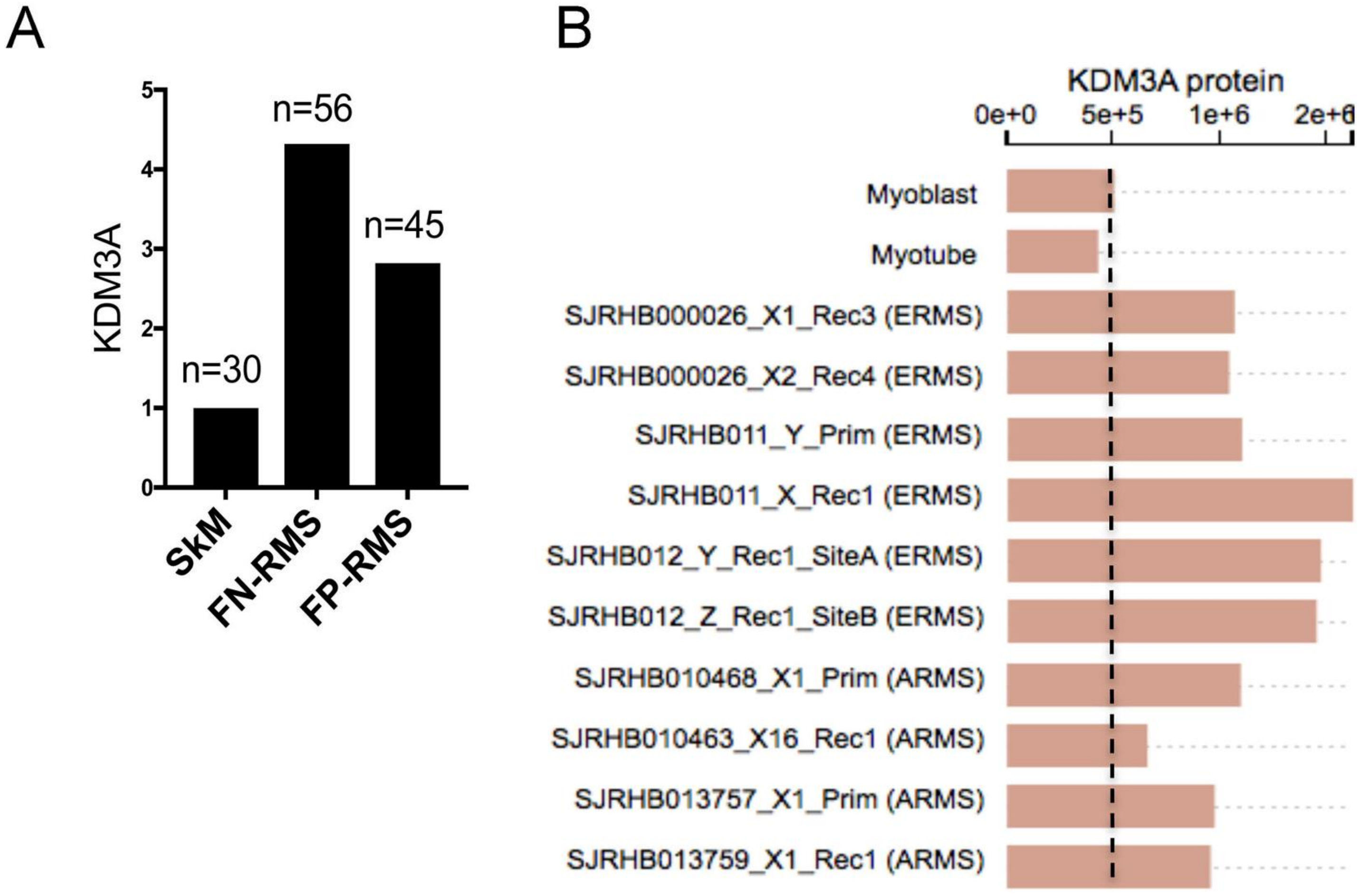

Supplementary Figure S1: KDM3A expression data from RMS patient tumors from Walters et al (RNA) [11] **A.,** and the St Jude Children's Research Hospital Pediatric Cancer (PeCan) database (https://pecan.stjude.cloud; protein) **B**.

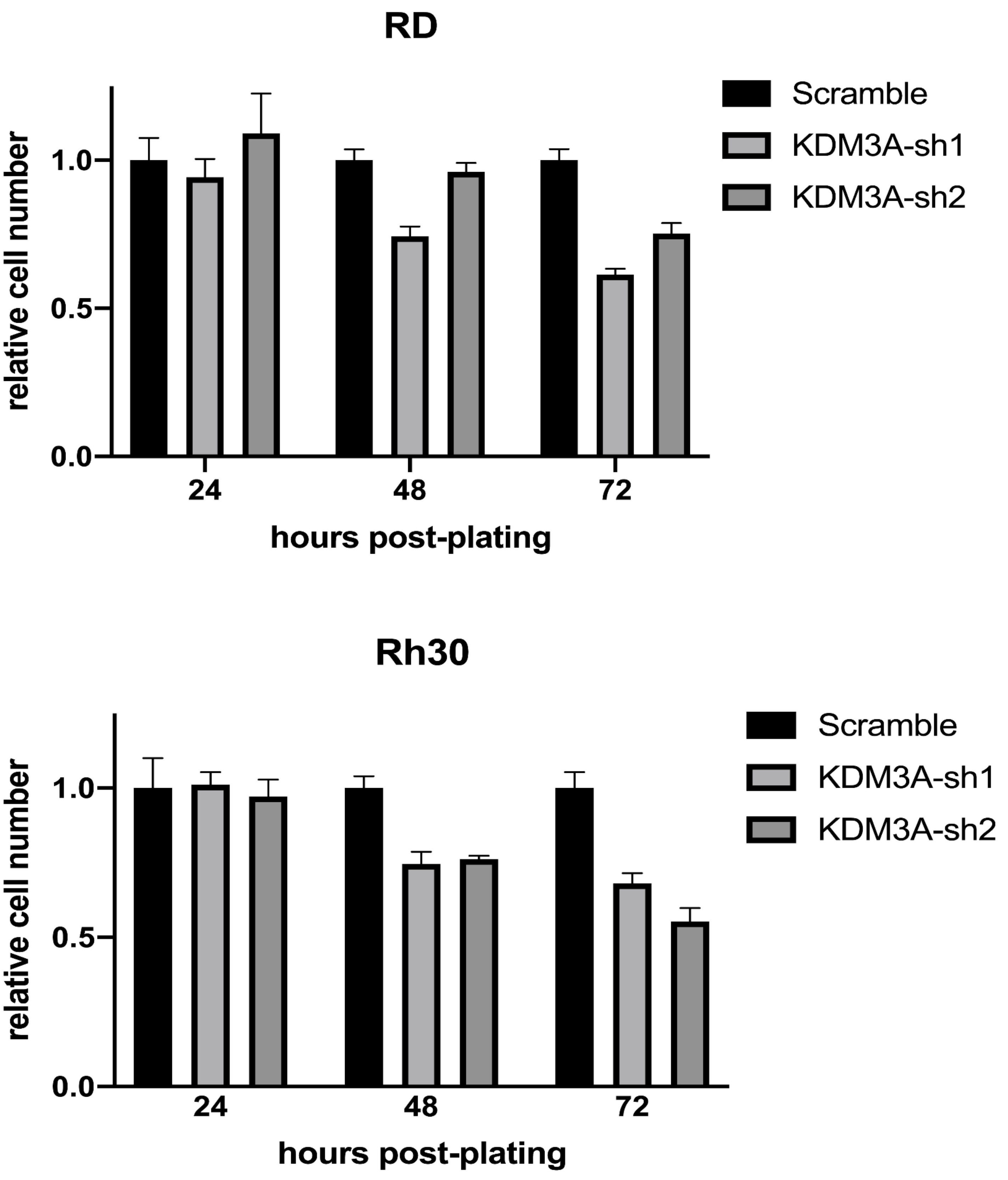

Supplementary Figure S2: Effects of KDM3A depletion on short-term cell growth/survival, as determined by MTT assays (mean and standard error of 2 independent experiments, each performed in at least triplicate (days 1 and 3); mean and standard deviation of 6 replicates (day 2)).

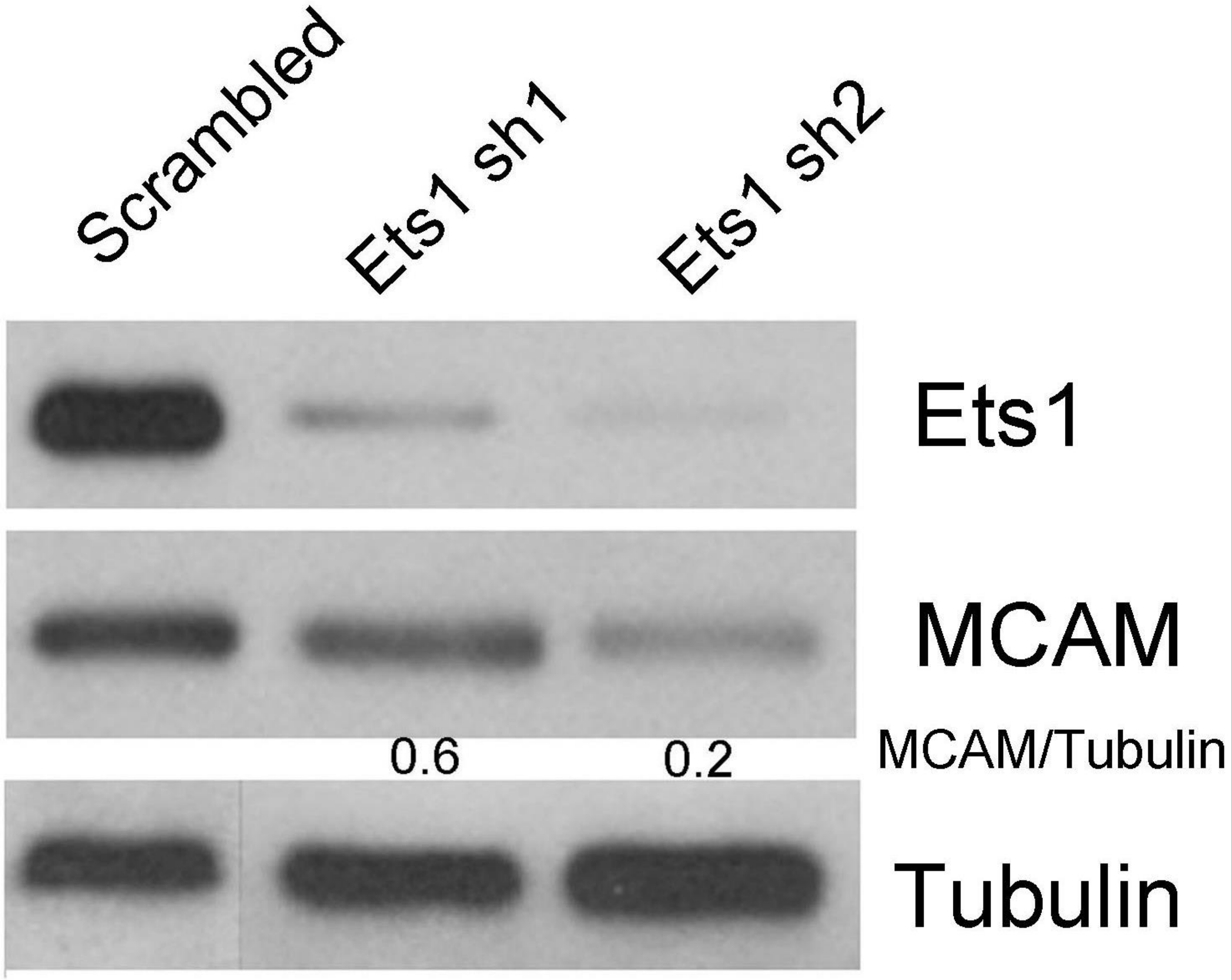

Supplementary Figure S3: Effect of Ets1 depletion on MCAM expression in Ras^{WT} FN-RMS Rh18 cells, as determined by immunoblotting (Scrambled control is from same gel and exposure, but a non-adjacent lane).

A

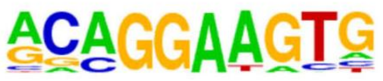

B

GGGGGT**GCGGAAGTT**GGAGAATTTTTCCAGAATGTGGGTGTGAGTAGGAGAAGATTATACTGAAGGAGTGAAAACA
GGGTGGAAAAGGGTAGGGGGCAGGAA**ACCGGATACT**GTACTGGGGGGTGGATGGAAGATGTGGGGGAAGAGATTG
AATCGGGGGAAGCAGCCCTGCTCCC**GCCGGATGCT**GTCTCACCCATGTCCTTCGCACCTGACCCAGGGCAGGAGGAAG
GAGGCACAGCCGGGATGGAGGTGCACCCCACACCACCATGGGAGAGAGGGAGGGTGGCCTTCTGCGGGATGGGAG
GGGCTATTTTTAAACTTGGAAAAACCGTGAGTTCATCTCCATTCTGGAGGCTGCAAAGGCCCCATTGTACAGGGGTCTA
GAGAGGGGGAGCGGGGGGAGGGGGCGACGGGCACAATGGCGAGGATTATGTTGGAGCAGGCAGGGGGGTGGAGG
GCGGAGAGCGGGGGAGGGGCTGTCTTCGTCACAGATGGCCTACTGAACTCTGGGGTCGAGCCAGGCTGGGGCTTGA
ACTGGGGGGCAGGGCCCACCCTCACAAAGATCCCCGTTGGGGAACTGGGGTCTCT**GACATCCGGT**GGCCCA

Supplementary Figure S4: **A.** Human Ets1 consensus DNA regulatory element (from http://homer.ucsd.edu/homer/motif/motifDatabase.html); **B,** sequence of genomic region upstream of MCAM gene (“EBS R” in Figure 3), containing four candidate Ets1 binding sites (bold blue).

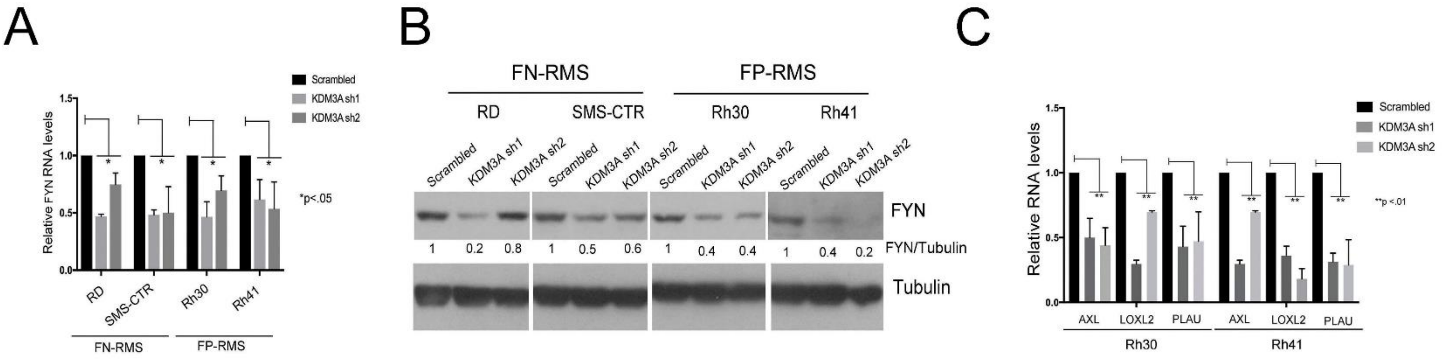

Supplementary Figure S5: **A.** Expression of FYN in control and KDM3A knockdown FN-RMS and FP-RMS cells, as determined using qRT-PCR (left panel) and immunoblotting (right panel). **B.** AXL, LOXL2 and PLAU expression in control and KDM3A knockdown FP-RMS cells as determined by qRT-PCR. P-values were determined using a two-way Student’s t-test with unequal variance.
